# A systematic review of differential rate of use of the word “evolve” across fields

**DOI:** 10.7717/peerj.3639

**Published:** 2017-08-21

**Authors:** Nina Singh, Matthew T. Sit, Marissa K. Schutte, Gabriel E. Chan, Jeyson E. Aldana, Diana Cervantes, Clyde H. Himmelstein, Pamela J. Yeh

**Affiliations:** Department of Ecology and Evolutionary Biology, University of California, Los Angeles, CA, United States of America

**Keywords:** Evolve, Resistance, Cancer, HIV, Mosquito, Weed, Science, Clinical, Fields

## Abstract

**Background:**

Although evolution is the driving force behind many of today’s major public health and agriculture issues, both journalists and scientific researchers often do not use the term “evolve” in discussions of these topics.

**Methods:**

In a total of 1,066 articles and 716 papers selected from 25 US newspapers and 34 scientific journals, we assess usage of the word “evolve” and its substitute words in the contexts of cancer tumor drug resistance, HIV drug resistance, mosquito insecticide resistance, and weed pesticide resistance.

**Results:**

We find significant differences in the use of “evolve” among fields and sources. “Evolve” is used most when discussing weed pesticide resistance (25.9% in newspapers, 52.4% in journals) and least when discussing cancer tumor drug resistance (3.9% in newspapers, 9.8% in journals). On average, scientific journals use “evolve” more often (22.2%) than newspapers (7.8%). Different types of journals (general science, general clinical, cancer specific, and drug resistance specific) show significantly different “evolve” usages when discussing cancer tumor drug resistance.

**Discussion:**

We examine potential explanations of these findings, such as the relatively recent framing of cancer in evolutionary terms, before looking at consequences of low “evolve” usage and of differential “evolve” usage across fields. Use of the word “evolve” may not reflect current understanding of the problems we examine. However, given that our ability to tackle resistance issues relies upon accurate understandings of what causes and exacerbates resistance, use of the word “evolve” when called for may help us confront these issues in the future.

## Introduction

The media can play an important role in the public’s awareness of evolutionary concepts (Antonovics et al., 2007; Ashgar, Wiles & Alters, 2007; Dingwall & Aldridge, 2006) and scientific journals may reflect current professional uses of evolutionary concepts across different fields (Antonovics et al., 2007). These sources provide opportunities to gauge how frequently the general public and scientists are exposed to the term “evolve,” and allow us to determine if there is significant variation in “evolve” usage across fields and focal organisms. For example, when journal abstracts discussing cancer relapse and therapeutic resistance were examined, only 1% of abstracts were found to use evolutionary language (Aktipis et al., 2011). Even in reference to antimicrobial resistance, which is one of the most widely accepted examples of evolution (Read & Huijben, 2009), the usage of the word “evolve” is low, at 33% in biomedical journal papers (Antonovics et al., 2007) and 18% in newspaper articles (Singh et al., 2016). Evolution of antibiotic resistance is often described using substitute words such as “emerge” or “develop,” instead of the more scientifically accurate word “evolve” (Antonovics et al., 2007; Singh et al., 2016). This raises the question of whether the discourse on other issues of resistance (e.g., pesticide resistance in weeds) is framed in evolutionary terms.

Use of the term “evolve” does not mean the concept of evolution is fully understood by the writer and reading a newspaper article or journal paper that includes the word “evolve” does not mean the reader will better understand evolution. However, using correct terminology—and specifically using the term evolution when it is called for—can be seen as a first step towards better explaining and understanding evolution. There are still low percentages of evolution acceptance in the US (Miller, Scott & Okamoto, 2006). A Pew Research survey from 2014 (Pew Research Center, 2015) indicates that only 35% of those surveyed believed that animals and other living things evolved over time. A 2010 study on natural history museum visitors suggests that even among those who believe that organisms evolve, a majority only recognize evolution in the context of certain species and timescales (Evans et al., 2010).

In addition to variability in recognition and acceptance of evolution within the general American public, there may be differences in the ways that different groups of scientists approach evolution. While it has been reported that the scientific community has higher rates of evolution acceptance than the general population (Wiles, 2010), there is variation among scientists in use of the word “evolve.” When discussing antibiotic resistance, evolutionary biologists have been found to use the word more frequently than biomedical researchers (Antonovics et al., 2007). This may reflect the observation that a majority of people who work on drug resistance, including microbiologists, clinicians, and public health practitioners, typically do not have significant formal training in evolutionary biology (Read & Huijben, 2009).

Low levels of recognition of evolution in the context of important issues are potentially concerning because of the health and economic costs associated with the evolution of resistance. Cancer cells can evolve resistance to forms of chemotherapy that may have previously been effective (Frank & Rosner, 2012), mosquito populations can evolve resistance to insecticides and continue to spread mosquito borne diseases (Liu, 2015), and approximately 250 species of weed pests have already evolved resistance to a total of 160 different herbicides, with expensive consequences (Heap, 2017; Schulz & Segobye, 2016). A conservative 2001 estimate of the annual “evolution bill” in the US ranges from $33 billion to $50 billion, and includes costs associated with such problems as agricultural losses due to pesticide resistance and the development of new HIV/AIDS medication for resistance-evolving viruses (Palumbi, 2001). These costs are only expected to increase (Palumbi, 2001).

Recognizing these problems as evolutionary issues allows the application of rigorous evolutionary theory to solving them (Nesse & Stearns, 2008). Widespread recognition of these issues as evolutionary problems would also help to address them because similar methods to combat resistance (e.g., cycling of herbicides in agriculture and drug cycling in hospitals) have been independently discovered in several fields (Palumbi, 2001). Describing resistance problems in evolutionary terms should help identify common solutions across fields.

In this study, we aim to raise awareness of lack of use of the word “evolve” when discussing biological issues of resistance evolution and to help determine whether there is a need for biologists to use more scientifically accurate terms in their studies. We examine use of the word “evolve” in written discussions of cancer tumor drug resistance, HIV drug resistance, mosquito insecticide resistance, and weed pesticide resistance. We examine newspaper articles in each of these contexts as well as scientific papers from journals that specialize in the following focus areas: general science, general clinical, cancer-specific, drug resistance, virology, entomology, and agronomy and crop science. We compare “evolve” usage to see if there are significant differences depending on source (newspaper or journal) and field (oncology, virology, entomology, agronomy).

We expect there to be considerable differences among fields and furthermore consider special properties of certain categories in our analysis. For instance, HIV has been historically more prominent in some cities than others, and thus we conduct an additional test comparing “evolve” usage to city of newspaper publication for the HIV drug resistance category. In addition, evolution in cancer tumor cells is different from evolution in the other sub-fields because it was recognized relatively recently (Nowell, 1976) and tumor cells evolve within an individual, while evolution is traditionally thought of as happening across individuals within a population over time (Crespi & Summers, 2005). We therefore considered an additional set of tests for tumors, to examine how the use of “evolve” to discuss tumor evolution has changed among different groups of cancer researchers over the decades since the idea was introduced. We compare “evolve” usage when discussing tumor resistance in different types of journals (general science, general clinical, cancer-specific, drug resistance). We finally look at how the use of “evolve” when discussing tumor resistance in newspapers, journals, and evolution textbooks has changed over time.

**Table 1:** List of newspapers examined. Quantities of relevant articles identified in each category, as well as in total, for each newspaper examined. An article is “relevant” if it contains phrase(s) in which it is reasonable to expect use of the word “evolve” in its corresponding context of resistance. Letters to the editor, articles with multiple disconnected topics unrelated to antibiotic resistance, articles that were less than 100 words, and repeated articles were excluded.

Newspaper	Cancer tumor resistance	HIV resistance	Weed pesticide resistance	Mosquito Insecticide Resistance	Number of Total Relevant Articles
Chicago Sun Times (Illinois)	5	20	0	0	25
Cleveland Plain Dealer (Ohio)	8	28	2	2	40
Dallas Morning News (Texas)	10	28	0	4	42
Denver Post (Colorado)	4	18	3	1	26
Honolulu Star Advertiser (Hawaii)	1	0	2	1	4
Houston Chronicle (Texas)	9	45	6	6	66
Los Angeles Investor’s Business Daily (California)	1	5	0	0	6
Los Angeles Times (California)	22	67	16	15	120
Miami Herald (Florida)	10	39	0	5	54
Minneapolis Star Tribune (Minnesota)	4	7	4	1	16
New York Daily News (New York)	4	8	0	0	12
New York Times (New York)	42	55	13	11	121
Newark Star Ledger (New Jersey)	9	68	0	2	79
Philadelphia Inquirer (Pennsylvania)	15	52	2	1	70
Riverside County Press Enterprise (California)	0	11	0	2	13
Salt Lake City Deseret News (Utah)	8	33	2	4	47
San Francisco Chronicle (California)	10	58	3	1	72
St. Paul Pioneer Press (Minnesota)	3	14	2	1	20
USA Today (Virginia)	8	31	12	0	51
Wall Street Journal (New York)	58	99	18	7	182

## Materials & Methods

Following the methods in Singh et al. (2016), we identified the top 25 US digital daily newspapers using the Alliance for Audited Media (Lulofs, 2013) and accessed them via Newsbank, LexisNexis, Proquest, and EBSCOhost (Table 1). We excluded newspapers that we either could not fully access using these University of California-subscribed databases or that did not contain articles relevant to our study. Authors MKS, GEC, JEA, DC, and CHH searched the phrases “cancer cell resistance,” “cancer drug resistance,” “tumor drug resistance,” “cancer tumor treatment failure,” “HIV resistance,” “mosquito insecticide resistance,” “plant pesticide resistance,” “weed pesticide resistance,” and “weed herbicide resistance” (excluding “evolve” from all terms to avoid biasing the search), then carefully read each article published between 1980 and 2015 to determine relevancy. If there were disagreements about relevancy, multiple authors discussed the article and MKS made the final decision. We classified articles as relevant if they contained at least one phrase in which it was reasonable to expect use of the word “evolve” in the context of the biological evolution of resistance in the context of the search term. If the article discussed resistance without explaining that it evolved, it was not considered relevant, as there was no discussion of the development of resistance in which use of the word “evolve” could be expected. We excluded letters to the editor, articles that were less than 100 words, articles that contained multiple disconnected topics unrelated to our search term, and duplicate articles. In the case of duplicate articles, the longer article (if one version had been published with more material) was chosen. If they were the same length, the more recent article was chosen because any word changes represented the most updated views of the author.

We used SCImago Journal and Country Rank (SCImago, 2007), which determines rank by the number of citations received by a journal and the prestige of the journal where the citation was published, to find the top five journals specific to each of the following categories accessed using the Melvyl University of California Catalog: oncology, virology, entomology, and agronomy and crop science (Table 2). We searched the phrases previously mentioned, limiting our search to 1980–2015 to match the newspaper year range, and then randomly selected 100 papers for each journal to check for relevancy, checking all papers in a journal when our keywords yielded fewer than 100 results. We used the same relevancy criteria that were used for the newspapers.

**Table 2:** List of journals examined. Category each journal examined was classified as and quantities of relevant papers identified for each journal. * indicates the Oncology journals identified through SCImago Journal and Country Rank. These journals were combined with similar major journals (indicated as Cancer-Specific without (*)) to form the Cancer-Specific category that was used for analysis. A paper is “relevant” if it contains phrase(s) in which it is reasonable to expect use of the word “evolve” in its corresponding context of resistance.

Category	Journal	Number of Relevant Papers
General Science	Current Biology	3
General Science	Nature	42
General Science	PLOS Biology	13
General Science	PNAS	20
General Science	Science	28
General Clinical	JAMA	7
General Clinical	Lancet	7
General Clinical	Mayo Clinic Proceedings	6
General Clinical	New England Journal of Medicine	32
General Clinical	The American Journal of Medicine	3
Cancer-Specific	Cancer Cell	67
Cancer-Specific*	Cancer Research	40
Cancer-Specific*	Clinical Cancer Research	53
Cancer-Specific	International Journal of Cancer	25
Cancer-Specific*	Journal of Clinical Oncology	15
Cancer-Specific	Journal of the National Cancer Institute	32
Cancer-Specific	Nature Reviews Cancer	46
Cancer-Specific*	PLOS Genetics	17
Drug Resistance	Drug Resistance Updates	43
Virology	Journal of General Virology	24
Virology	Journal of Virology	41
Virology	PLOS Pathogens	25
Virology	The American Journal of Tropical Medicine and Hygiene	1
Virology	Virology	35
Agronomy and Crop Science	Agriculture, Ecosystems, and Environment	7
Agronomy and Crop Science	Biomass and Bioenergy	0
Agronomy and Crop Science	Industrial Crops and Products	0
Agronomy and Crop Science	Journal of the Science of Food and Agriculture	1
Agronomy and Crop Science	Theoretical and Applied Genetics	13
Mosquito	Annual Review of Entomology	4
Mosquito	Journal of Economic Entomology	40
Mosquito	Journal of Insect Physiology	3
Mosquito	Pest Management Science	21
Mosquito	The Journal of Experimental Biology	2

We further identified different categories of journals that published papers on cancer to examine “evolve” usage by different groups of tumor resistance researchers. We selected widely known journals with high impact factors: five general science journals, five general clinical journals, five cancer-specific journals, and a journal dedicated to drug resistance (Table 2). We applied the same methods as for other journals to select relevant papers.

Once we collected the relevant newspaper articles (Table S1) and papers (Table S2), we searched for all of the lexemes of the word “evolve” (e.g., “have evolved,” “evolves,” etc.) by using “evol” in the browser search feature and carefully reading the surrounding text to confirm that the word was used in the context of the evolution of resistance. We counted the number of times each article used “evolve” in the context of the search term used in order to determine if there was significant variation. Using the same method, we searched for the substitute words “acquire,” “adapt,” “develop,” “emerge,” and “mutate” and their lexemes. This list of substitute words does not include all possible cases, including some near-synonyms, because we chose these substitute words based on those found in previous studies (Antonovics et al., 2007; Singh et al., 2016) and a preliminary survey of the data. Multiple readers assessed each article to confirm relevancy and that “evolve” and the substitute words were used in the appropriate context. We note that not all substitute words were incorrect—for example, “mutate” could be as appropriate as “evolve” in specific contexts and close reading for context-dependence was therefore an important component of our methods.

To report “evolve” usages for a given category (e.g., HIV resistance newspaper articles), we divided the number of relevant articles that used “evolve” by the total number of relevant articles. The *p*-values for comparing all proportions were computed using the chi square test. Correlations were conducted using the Pearson product-moment correlation. We combined the oncology journals and the additional cancer-specific journals we selected in a single category: cancer-specific (Table 2). We used this category to represent cancer journals for comparisons between categories, and to compare against other categories of cancer journals: general science, general clinical, and the drug resistance specific journal.

We also compared our data on newspaper “evolve” usage and number of relevant articles in the HIV drug resistance category with geographic relevance. We compared each newspaper’s HIV drug resistance “evolve” frequency with the HIV incidence rate indicated in the state that its headquarters resided in as reported in the Centers of Disease Control and Prevention’s 2014 HIV Surveillance Report (CDC, 2015). In the case of multiple newspapers from the same state, the total number of instances of “evolve” usage was divided by the total number of relevant HIV articles examined from these newspapers to compute a weighted, representative frequency.

Lastly, in order to better understand how recently appreciated the cancer tumor evolution phenomenon is, we determined the “evolve” frequencies when discussing this phenomenon for both newspapers and journals for each year that we collected data for (1980–2015) and tested for a trend in usage over time. Furthermore, we examined modern textbooks to determine how widely implemented this phenomenon is. We filtered textbooks by “United States” and “biology” and then the search term “evolution” using The Open Syllabus Project (The American Assembly, 2016), which determines which textbooks are most popularly used in publically-accessible college syllabi. We looked for cancer in the indexes of the top 10 textbooks that were published after 1976 (when the paper first introducing tumor evolution (Nowell, 1976) came out) and only analyzed textbooks with titles that suggested a scope of general/introductory evolution or molecular evolution (Table S3).

## Results

**Figure 1:**
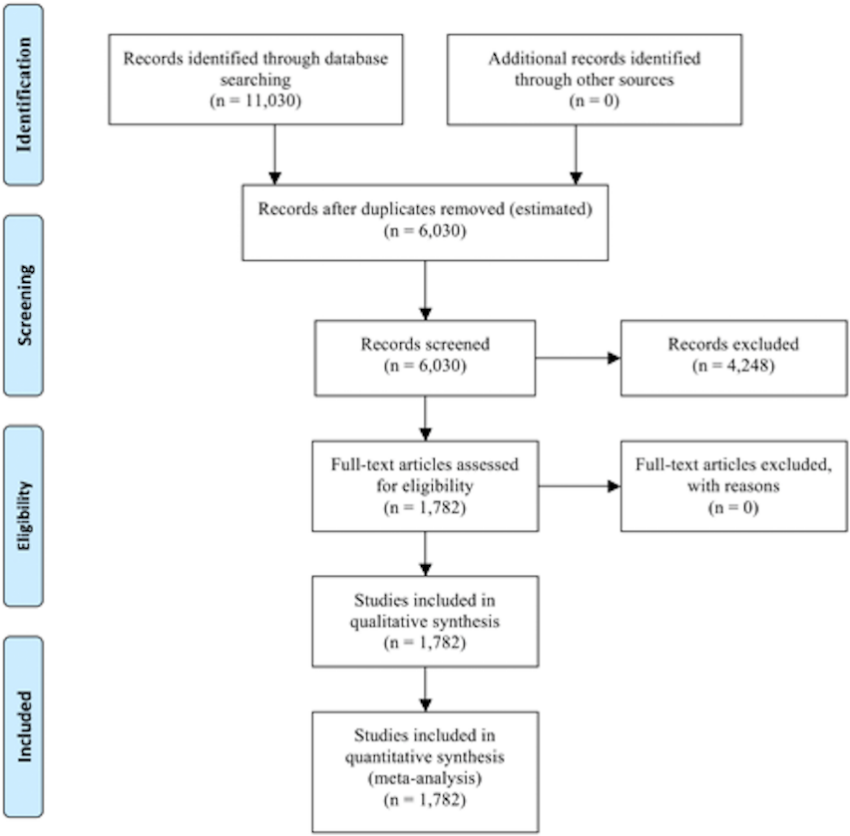
PRISMA flow diagram describing study records. The number of articles (for all newspapers and journals combined) at each stage of the screening process is shown.

**Figure 2:**
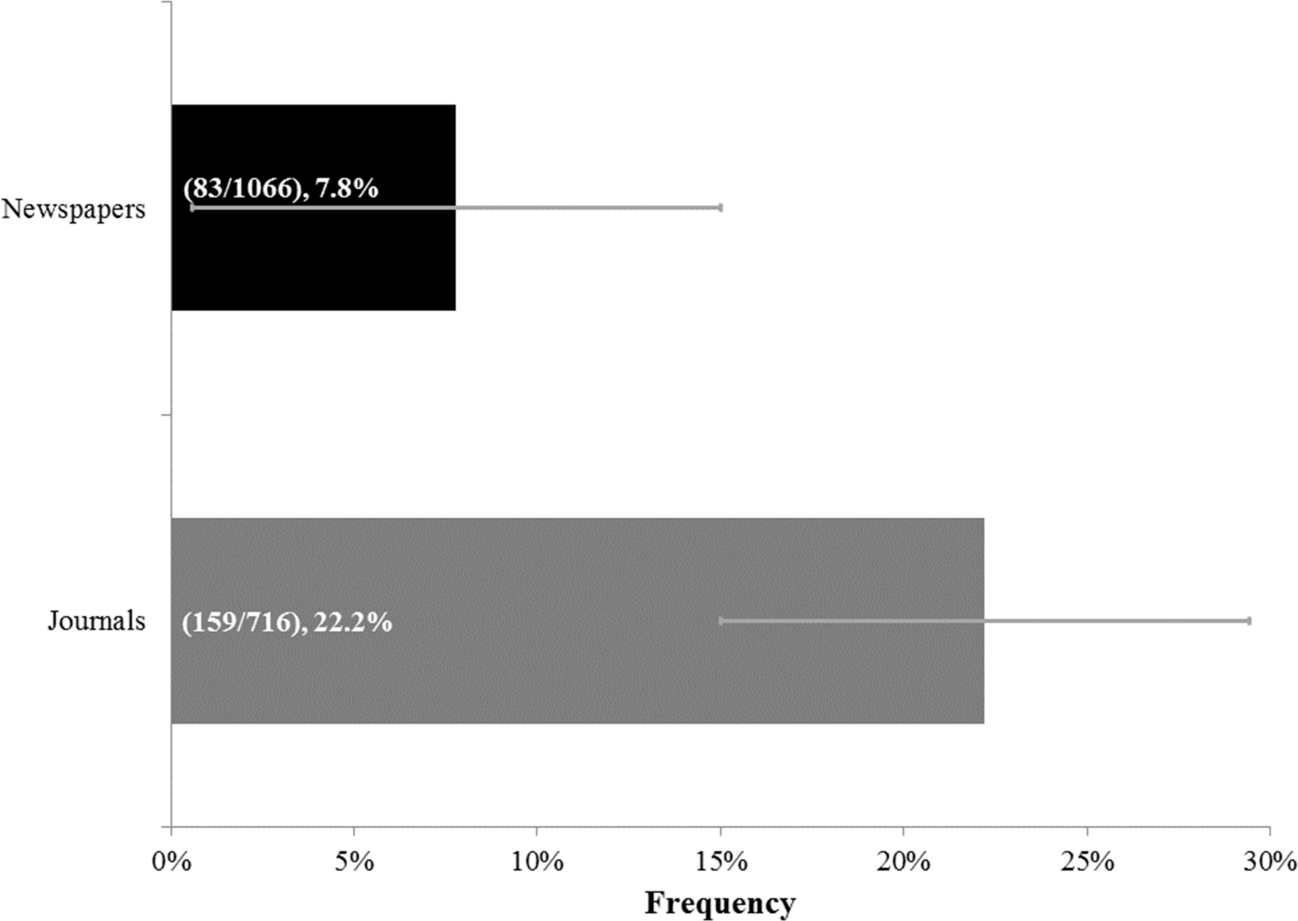
Frequencies of “Evolve” usage across newspapers and journals overall. All newspaper articles and journal papers examined are included in this figure. Parenthetical bar labels indicate: (quantity of relevant articles or papers using “evolve”/quantity of relevant articles or papers examined). Standard errors are shown.

**Figure 3:**
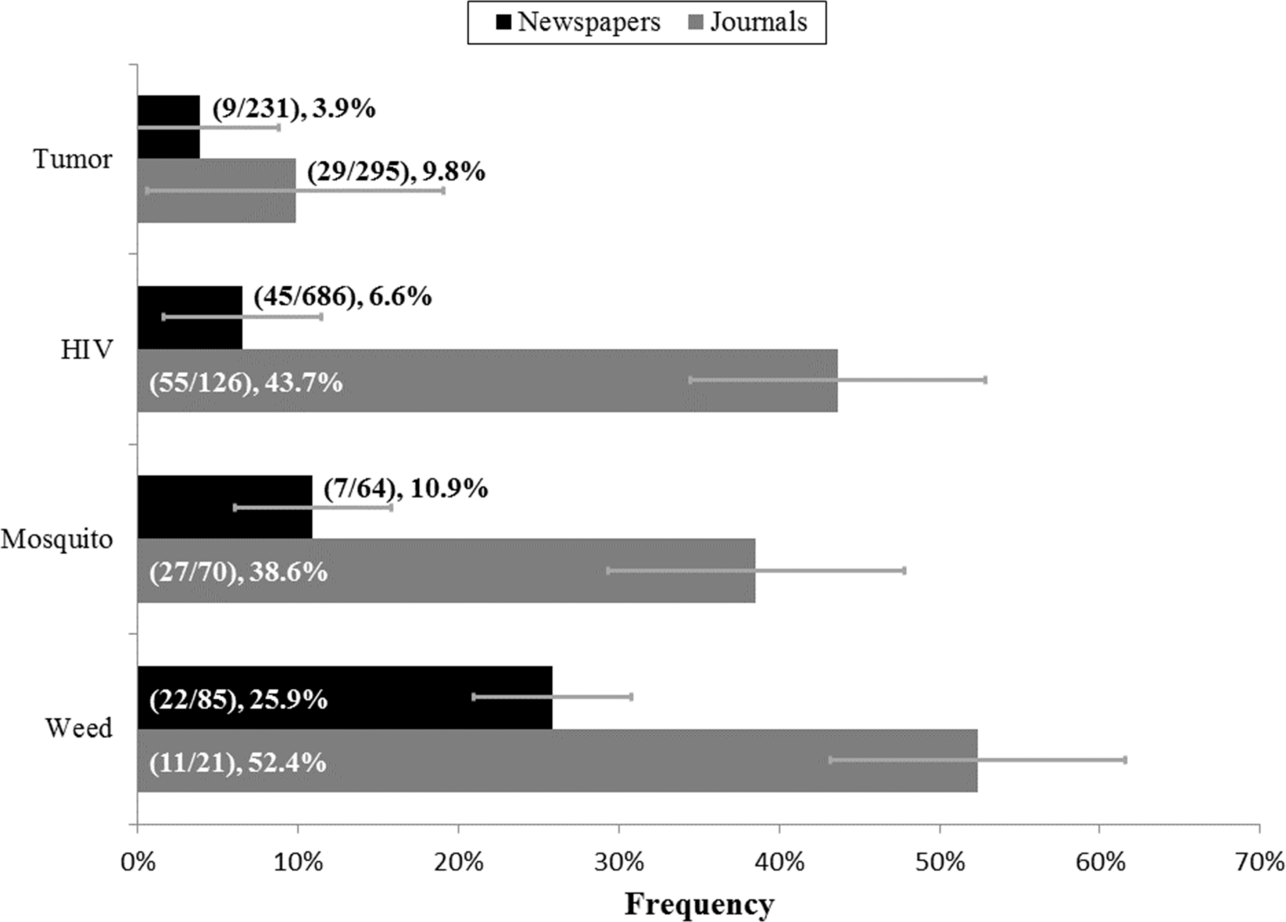
Frequencies of “Evolve” usage across newspaper and journal categories. Grouped by relevant category of resistance. Papers corresponding to the journal categories of “General Science,” “General Clinical,” and “Drug Resistance” are excluded from this figure. Parenthetical bar labels indicate: (quantity of relevant articles or papers using “evolve”/quantity of relevant articles or papers examined). Standard errors are shown.

The number of articles identified during each stage of the screening process is shown in Fig. 1. For the articles examined, we found that overall “evolve” usages in articles dealing with resistance were 7.8% in newspaper articles and 22.2% in scientific journal papers (Fig. 2), which represented a significant difference (*p*-value < 0.00001). With regards to specific categories (tumor, HIV, mosquito, weed) for newspapers and journals, “evolve” usage ranged from 3.9% to 52.4% (Fig. 3). There was, however, a significant difference between the frequencies of “evolve” usage across each category for both newspapers (*p*-value < 0.00001) and journals (*p*-value < 0.00001). For newspapers, weed pesticide resistance had the highest “evolve” usage frequency with 25.9%, followed in order by mosquito, HIV, and tumor resistance. For journals, the weed pesticide resistance category again used “evolve” at the highest levels at 52.4%, followed in order by HIV, mosquito, and tumor resistance (Fig. 3).

**Figure 4:**
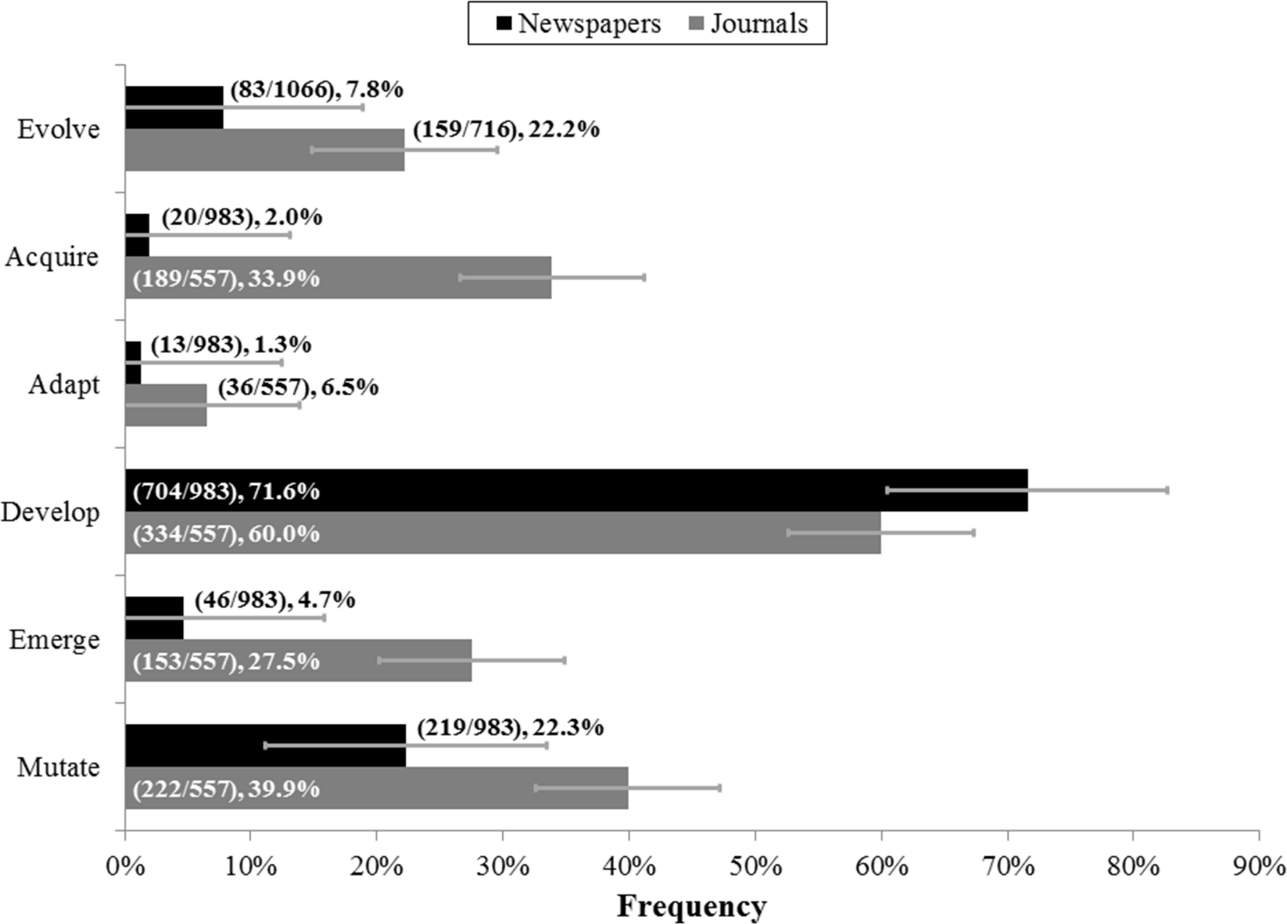
Frequencies of substitute word usage across newspapers and journals. Alternative words utilized by newspaper articles and journal papers that fail to use the word “evolve” (and thus these frequencies are calculated out of fewer items than those of “evolve,” which are out of the total number of relevant items). “Evolve” usage is also provided for reference. Parenthetical bar labels indicate: (quantity of relevant articles or papers using corresponding substitute word / quantity of relevant articles or papers examined). Standard errors are shown.

**Figure 5:**
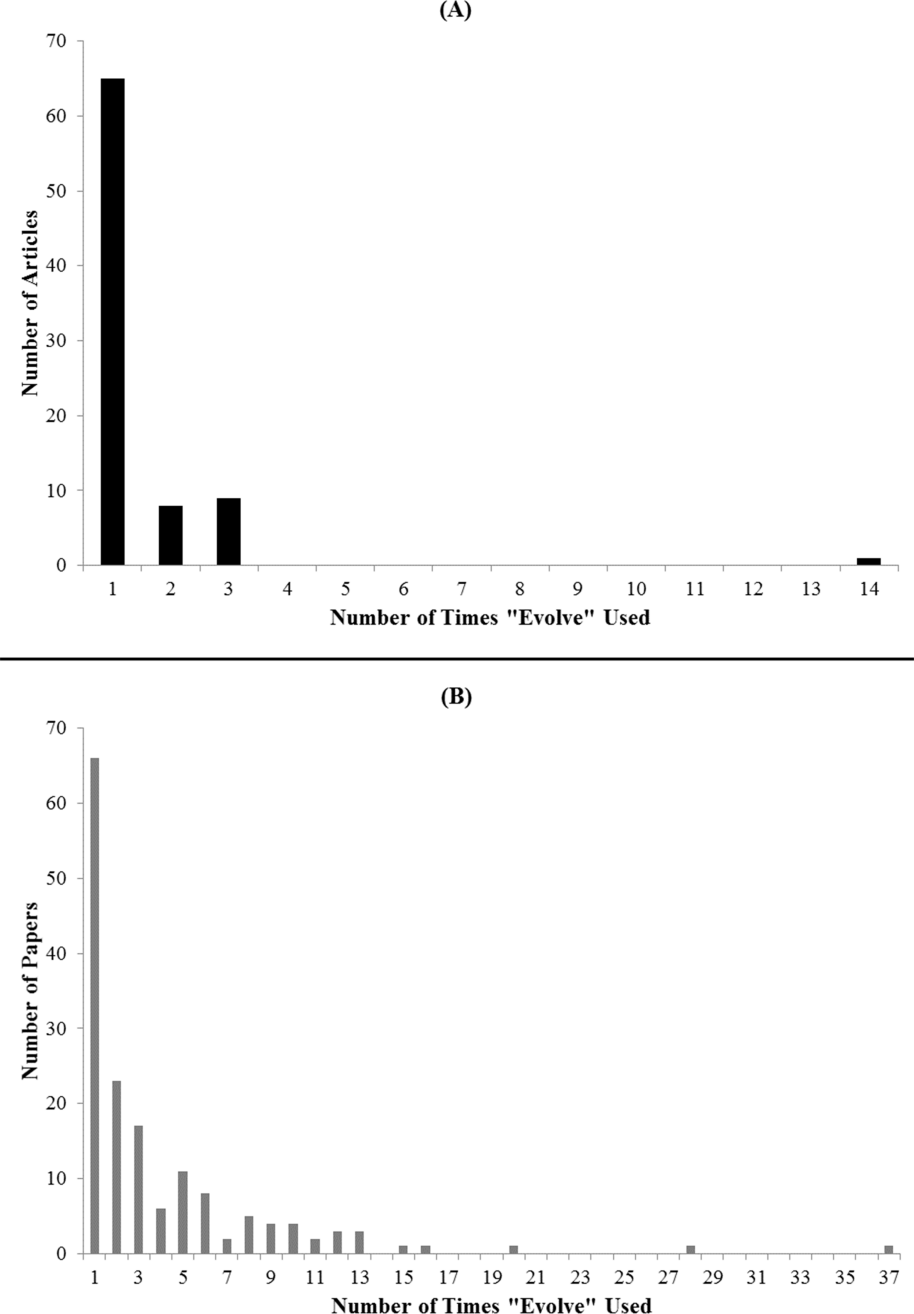
Number of times “Evolve” used in articles and papers that used “Evolve.” Bars shown depict the number of times “evolve” was used in the context of the search term (e.g., “weed pesticide resistance”) used to find the article or paper. Only articles and papers in which “evolve” was used at least once are shown.

We found that in both newspapers and journals, the substitute words “develop” and “mutate” were used much more frequently than the word “evolve” (Fig. 4). Additionally, while newspapers mostly used only these two words more than “evolve,” journals also used the substitute words “emerge” and “acquire” more often than “evolve.” When we examined the number of “evolve” usages in newspapers and journals, we found that there was not much variation (Fig. 5).

**Figure 6:**
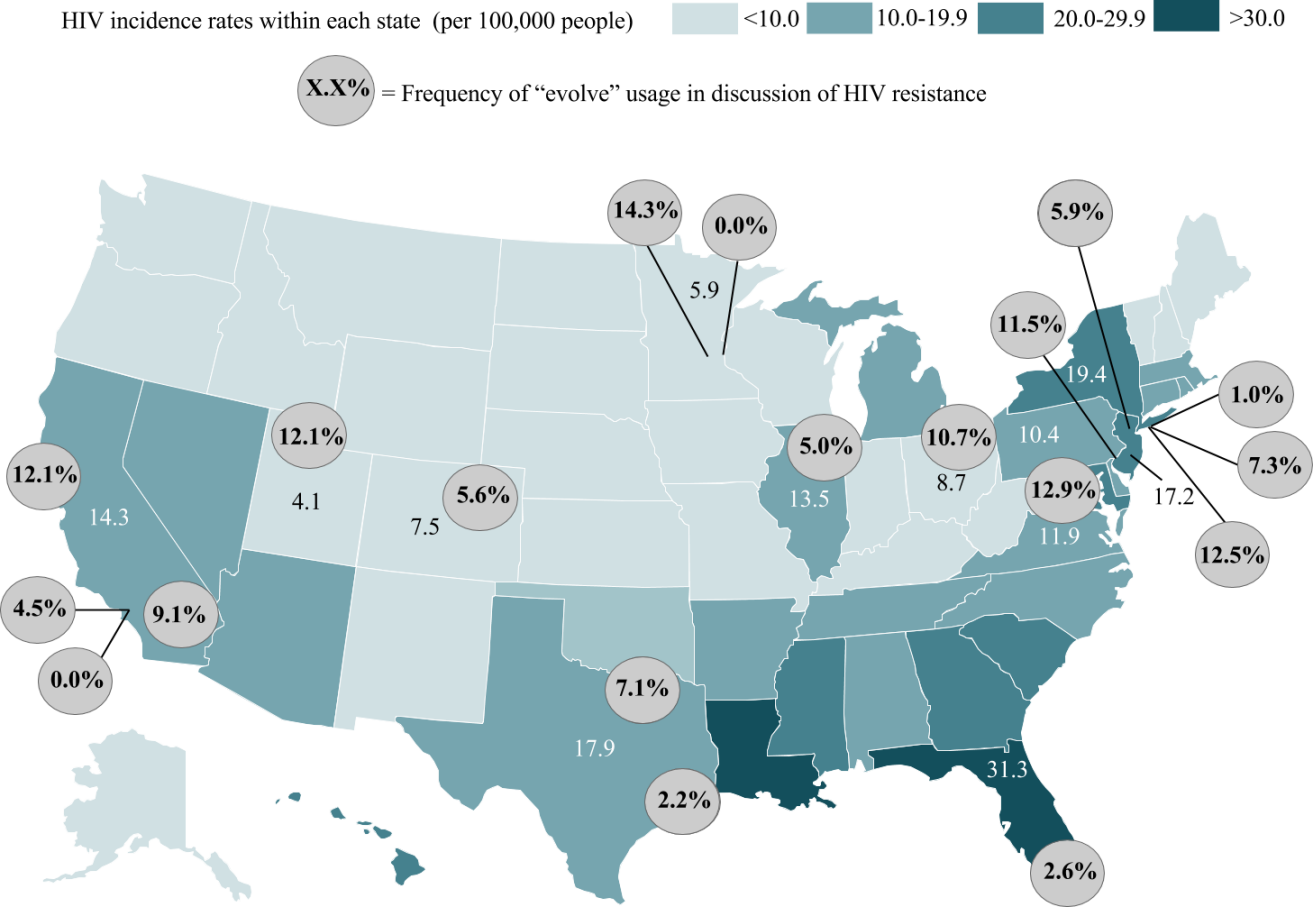
Percent “Evolve” usage in newspapers and HIV incidence across the United States. Values in circles display the percentage of relevant articles that used the word “evolve” in articles discussing HIV resistance and are positioned over or around the city in which each newspaper is headquartered. The values embedded into the map display HIV diagnosis rates per 100,000 people as reported by the CDC in 2014. The *Honolulu Star Advertiser* is excluded from the map, as no relevant articles were found.

In associating the number of relevant articles and “evolve” usage frequency found within the HIV drug resistance category with HIV incidence rates per state, we did not find a significant correlation between number of relevant articles and HIV incidence rates (Pearson’s *r*: 0.44; *p*-value: 0.16) or between “evolve” usage and HIV incidence rates (Pearson’s *r*: −0.61; *p*-value: 0.04) (Fig. 6).

**Figure 7:**
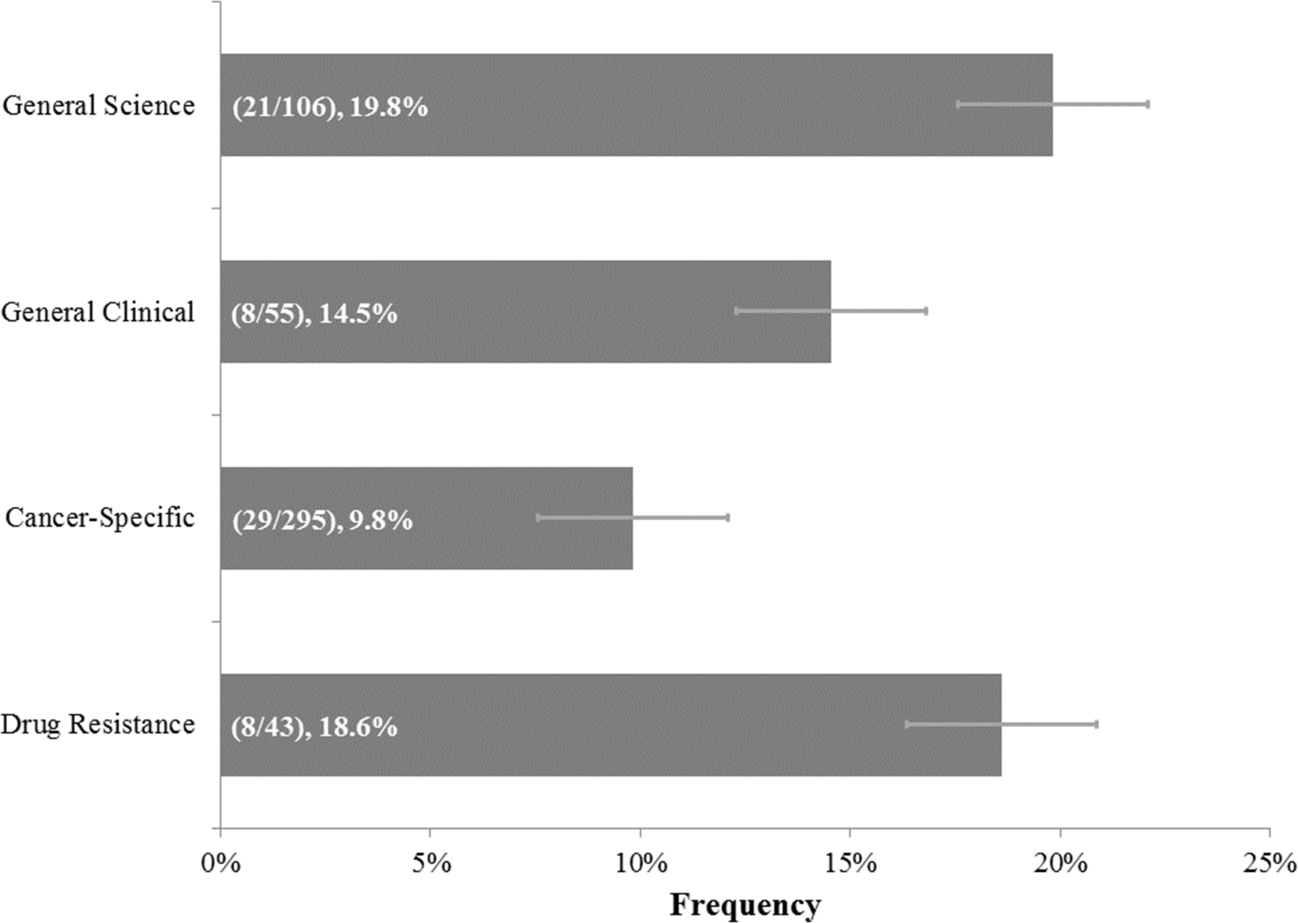
Frequencies of “Evolve” usage across cancer journal categories. Concerning journal papers relevant to “Cancer Tumor Drug Resistance” and of the “General Science,” “General Clinical,” “Cancer-Specific,” or “Drug Resistance” category. Parenthetical bar labels indicate: (quantity of relevant papers using “evolve”/quantity of relevant papers examined). Standard errors are shown.

Concerning the cancer tumor drug resistance category, both newspapers and journals used the word “evolve” less frequently in the context of cancer than all of the other categories combined (*p*-value: 0.01 for newspapers and *p*-value <0.00001 for journals) (Fig. 3). Moreover, across the different types of cancer journals, “evolve” usage also differed significantly (*p*-value: 0.04), with General Science and Drug Resistance category journals using “evolve” with the highest rates at 19.8% and 18.6%, respectively (Fig. 7).

**Figure 8:**
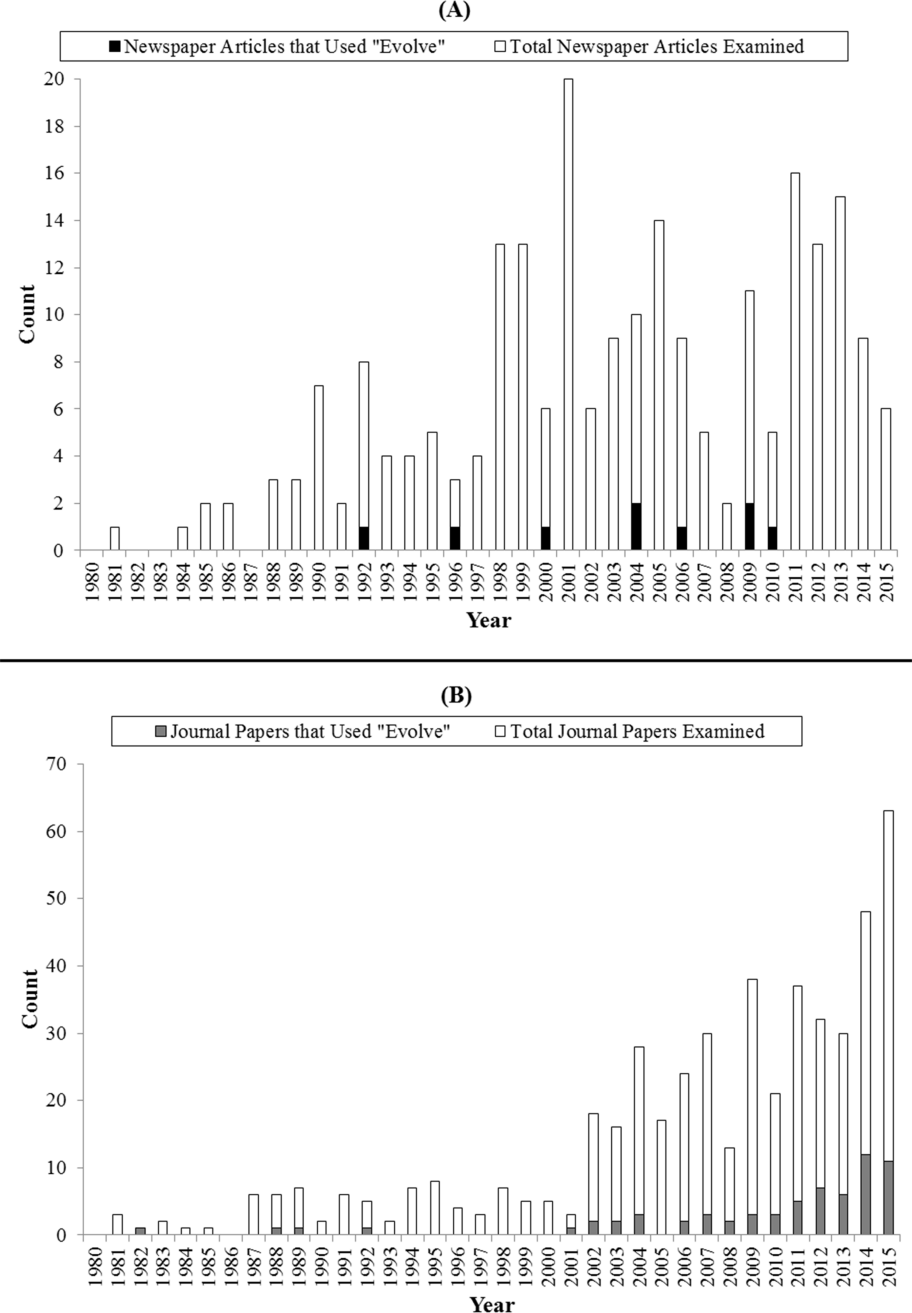
“Evolve” usage in cancer (A) newspaper and (B) journal categories. Cancer journal data includes the union of all papers categorized as “General Science,” “General Clinical,” “Cancer-Specific,” and “Drug Resistance.” Bars representing total items examined are shown as including both the shaded and unshaded segments of the bar.

Neither newspapers nor journals showed any tendency for “evolve” usage to increase over time for tumor resistance (Pearson’s *r*: 0.14; *p*-value: 0.43 and Pearson’s *r*: 0.03; *p*-value: 0.87, respectively) (Fig. 8), perhaps because overall usage is so low. Finally, we found that 2 out of the 10 evolution textbooks we examined mentioned “cancer” in the index and discussed it in a relevant evolutionary context.

## Discussion

Significant differences in the use of the word “evolve” within different types of scientific journals suggest that the use of evolutionary principles in discussing key issues may vary significantly between fields. Newspapers and journals both used the word “evolve” with different frequencies depending on the topic discussed (Fig. 3). Interestingly, both newspapers and journals used “evolve” most frequently when discussing pesticide resistance in weeds, and least frequently when discussing cancer tumor drug resistance. This may reflect the fact that reporters speak to and read various sources outside of scientific research when writing articles, including doctors, politicians, and industry professionals. These sources also influence the way that reporters choose to frame discourse on the issue (Berkowitz, 2009).

We expected that the newspapers from states with higher incidences of HIV would contain more articles about HIV drug resistance and perhaps a higher frequency of “evolve” usage than states with lower incidences. This is due to the potentially increased interest in understanding the development of viral resistance to drug therapies in areas where HIV incidence is high. However, our analysis revealed no correlation between number of relevant articles and HIV incidence, or between “evolve” usage and HIV incidence. In fact, “evolve” usage was low across all states (Fig. 6). A 2003 survey revealed that 72% of the US public reported that the media played a major role in providing them with important information concerning HIV/AIDS. This underscores the media’s role in providing information and education to the public, suggesting that the audience may not be well exposed to the role evolution plays in viral resistance (Mollyann et al., 2004).

It is important to note that the problem of word choice is a linguistics issue that has been examined from a biological perspective. The field of linguistics has developed many techniques to create text corpora to analyze, and although we were able to create a corpus of articles and papers and discuss its principles, we did not use linguostatistics to address corpus linguistics. While this analysis is less sophisticated, we hope that our results are able to raise awareness of lack of usage of the word “evolve” in the biological community and general public.

Simply using the word “evolve” does not indicate that the audience reading the article will understand evolution. Rather, we suggest that overall usage of the word “evolve” (for example, in a field or journal) can be a rough indicator of overall recognition of evolutionary processes. Use of “evolve” in newspapers and journals informs us about the way discussions about certain topics are framed rather than reflecting understanding or acceptance of those topics. Indeed, there is likely a large gap between how academics understand evolution and how the general public understands evolution (Nadelson & Sinatra, 2010; Kahlor & Stout, 2010; Spiegel et al., 2006). The science-society relationship is complex and does not rely solely on a top-down flow of information. It is complicated by multiple factors determining the way that the public reacts to this information (Kahlor & Stout, 2010).

But regardless of this gap between the general public and scientific audiences, we found that the word “evolve” was used significantly less frequently when discussing cancer tumor drug resistance than all other topics in both newspapers and journals (3.9% and 9.8%, respectively) (Fig. 3). A previous study examining the use of evolutionary language in journal abstracts discussing cancer relapse and therapeutic resistance reported even lower levels of “evolve” usage. They found that “evolve” usage has been at about 1% since the 1980’s and has increased only slightly in recent years (Aktipis et al., 2011).

Importantly, the idea of cancer tumor cells evolving in response to drugs is fairly new. While the scientific idea that cancer cells evolve was presented in 1976 (Nowell, 1976), the idea has only slowly taken hold (Aktipis et al., 2011). This is evidenced by the fact that in 1992, when 30 evolution textbooks were examined, not a single one mentioned tumor evolution (Graham, 1992). When we examined 10 evolution textbooks in the present day, only two mentioned tumor cells evolving. Furthermore, we found no trend of increasing usage of “evolve” over time in newspaper or journal cancer categories (Pearson correlations, *p*-values: 0.43 and 0.87, respectively). Together, these findings demonstrate that in the field of cancer, there has consistently been an overall low rate of discussions that include evolutionary concepts.

Our finding that “evolve” was used in the drug resistance journal and general science journals significantly more often than in the general clinical and cancer-specific journals (Fig. 7) could further reflect how evolutionary concepts are infrequently introduced and slow to spread in the medical field (Antonovics et al., 2007; Nesse & Stearns, 2008). While ecology and evolution journals cite medical journals occasionally, medical journals cite ecology and evolution journals rarely. This suggests an asymmetry in the relationship between medicine and evolutionary biology (Nesse & Stearns, 2008; Rosvall & Bergstrom, 2008). If healthcare professionals are less exposed to evolutionary principles, it could prevent them from using evolutionary perspectives in understanding and dealing with problems such as cancer tumor drug resistance. This is an area for investigation.

Indeed, the lower rate of “evolve” usage we found in journals with a more medical audience (general clinical and cancer-specific) may be reflective of a lack of recognition of evolution as an important concept for medical consideration. Most medical schools do not have evolutionary biologists on the faculty (Nesse & Stearns, 2008), and many medical students do not accept the theory of evolution (Nesse & Stearns, 2008). This may be due to the common conception that a clinician’s knowledge of the origins and evolution of pathogens is often not essential in administering successful treatments to patients (MacCallum, 2007), or it could be due to other reasons that we currently do not understand. However, increasing exposure to evolutionary concepts can be valuable in the overarching treatment of medical problems (Nesse et al., 2010; Nesse & Stearns, 2008; Stearns, 2012; Stearns et al., 2010). Not only can research on the evolutionary reasons behindclinical occurrences potentially help shape treatment options, but evolution can provide a framework for organizing medical knowledge more generally (Nesse & Stearns, 2008).

## Conclusions

Ultimately, although researchers demonstrate a greater tendency to use evolutionary language compared with journalists, the use of the word “evolve” was consistently low for both groups, especially in comparison to substitute words (Fig. 4). It is impossible to know *a priori* what word is more accurate in all possible contexts and the issue of choosing which word to use is more complex than we have described. However, the finding that “evolve” is used so rarely across fields and sources when describing the evolution of resistance is surprising. Furthermore, different frequencies of “evolve” usage depending on category (HIV drug resistance, tumor resistance, etc.) and type of journal discussing cancer tumor drug resistance could reflect discrepancies in recognition of resistance problems as evolutionary issues across fields (Antonovics et al., 2007). These discrepancies could be preventing certain fields from applying evolutionary theory to combat resistance issues or from recognizing the applicability of solutions to analogous issues in other fields (Palumbi, 2001).

There may be many reasons for why an author does not use the word “evolve.” However, using the word “evolve” instead of unclear or less accurate alternatives when discussing important evolutionary issues may help make the topic clearer to readers. In addition to increasing exposure to the theory of evolution, which may be downplayed due to low public acceptance (Scott & Branch, 2003), using the word “evolve” when discussing topics like tumor resistance and weed pesticide resistance may help shape the way that the general public, clinicians, and scientists in different fields think about and approach these topics.

##  Supplemental Information

10.7717/peerj.3639/supp-1 Supplemental Information 1 PRISMA flow diagram

10.7717/peerj.3639/supp-2 Supplemental Information 2 PRISMA checklist

10.7717/peerj.3639/supp-3 Table S1 Newspaper articles used in analysis

10.7717/peerj.3639/supp-4 Table S2 Journal papers used in analysis

10.7717/peerj.3639/supp-5 Table S3 Textbooks used in analysis

## Additional Information and Declarations

### Data Availability

The following information was supplied regarding data availability: The raw data has been supplied as Supplemental Files.
