# Supplementary material for: A systematic review of differential rate of use of the word “evolve” across fields
Source: PeerJ. 2017 Aug 21;5:e3639. doi: 10.7717/peerj.3639 (PMC5572546; doi:10.7717/peerj.3639)
Supplement: Supplemental Information 1 [file peerj-05-3639-s001.doc]

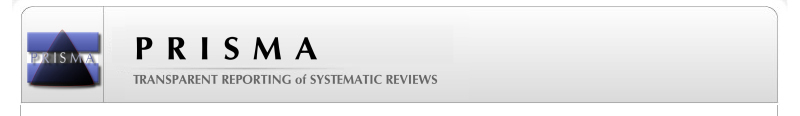
**PRISMA 2009 Flow Diagram**

**Screening**

**Included**

**Eligibility**

**Identification**

Records identified through database searching
(n = 11,030)

Additional records identified through other sources
(n = 0)

Records after duplicates removed (estimated)
(n = 6,030)

Records screened
(n = 6,030)

Records excluded
(n = 4,248)

Full-text articles assessed for eligibility
(n = 1,782)

Full-text articles excluded, with reasons
(n = 0)

Studies included in qualitative synthesis
(n = 1,782)

Studies included in quantitative synthesis (meta-analysis)
(n = 1,782)
