# Supplementary material for: A systematic review of differential rate of use of the word “evolve” across fields
Source: PeerJ. 2017 Aug 21;5:e3639. doi: 10.7717/peerj.3639 (PMC5572546; doi:10.7717/peerj.3639)
Supplement: Supplemental Information 2 [file peerj-05-3639-s002.docx]

| **Section/topic** | **#** | **Checklist item** | **Reported on page #** |
| --- | --- | --- | --- |
| **TITLE** | | |  |
| Title | 1 | Identify the report as a systematic review, meta-analysis, or both. | 1  “Differential rate of use of the word “evolve” across fields” |
| **ABSTRACT** | | |  |
| Structured summary | 2 | Provide a structured summary including, as applicable: background; objectives; data sources; study eligibility criteria, participants, and interventions; study appraisal and synthesis methods; results; limitations; conclusions and implications of key findings; systematic review registration number. | 2  **“Background.** Although evolution is the driving force behind many of today’s major public health and agriculture issues, both journalists and scientific researchers often do not use the term “evolve” in discussions of these topics.  **Methods.** In a total of 1066 articles and 716 papers from 25 U.S. newspapers and 34 scientific journals, we assess usage of the word “evolve” and its substitute words in the contexts of cancer tumor drug resistance, HIV drug resistance, mosquito insecticide resistance, and weed pesticide resistance.  **Results.** We find significant differences in the use of “evolve” among fields and sources. “Evolve” is used most when discussing weed pesticide resistance (25.9% in newspapers, 52.4% in journals) and least when discussing cancer tumor drug resistance (3.9% in newspapers, 9.8% in journals). On average, scientific journals use “evolve” more often (22.2%) than newspapers (7.9%). Different types of journals (general science, general clinical, cancer specific, and drug resistance specific) show significantly different “evolve” usages when discussing cancer tumor drug resistance.” |
| **INTRODUCTION** | | |  |
| Rationale | 3 | Describe the rationale for the review in the context of what is already known. | 3  “Even in reference to antimicrobial resistance, which is one of the most widely accepted examples of evolution (Read & Huijben 2009), the usage of the word “evolve” is low, at 33% in biomedical journal papers (Antonovics et al. 2007) and 18% in newspaper articles (Singh et al. 2016). Evolution of antibiotic resistance is often described using substitute words such as “emerge” or “develop,” instead of the more scientifically accurate word “evolve” (Antonovics et al. 2007; Singh et al. 2016). This raises the question of whether the discourse on other issues of resistance (e.g. pesticide resistance in weeds) is framed in evolutionary terms.” |
| Objectives | 4 | Provide an explicit statement of questions being addressed with reference to participants, interventions, comparisons, outcomes, and study design (PICOS). | 5-6  “In this study, we examine use of the word “evolve” in written discussions of cancer tumor drug resistance, HIV drug resistance, mosquito insecticide resistance, and weed pesticide resistance. We examine newspaper articles in each of these contexts as well as scientific papers from journals that specialize in the following focus areas: general science, general clinical, cancer-specific, drug resistance, virology, entomology, and agronomy and crop science. We compare “evolve” usage to see if there are significant differences depending on source (newspaper or journal) and field (oncology, virology, entomology, agronomy).  We expect there to be considerable differences among fields and furthermore consider special properties of certain categories in our analysis. For instance, HIV has been historically more prominent in some cities than others, and thus we conduct an additional test comparing “evolve” usage to city of newspaper publication for the HIV drug resistance category. In addition, evolution in cancer tumor cells is different from evolution in the other sub-fields because it was recognized relatively recently (Nowell 1976) and tumor cells evolve within an individual, while evolution is traditionally thought of as happening across individuals within a population over time (Crespi & Summers 2005). We therefore considered an additional set of tests for tumors, to examine how the use of “evolve” to discuss tumor evolution has changed among different groups of cancer researchers over the decades since the idea was introduced. We compare “evolve” usage when discussing tumor resistance in different types of journals (general science, general clinical, cancer-specific, drug resistance). We finally look at how the use of “evolve” when discussing tumor resistance in newspapers, journals, and evolution textbooks has changed over time.” |
| **METHODS** | | |  |
| Protocol and registration | 5 | Indicate if a review protocol exists, if and where it can be accessed (e.g., Web address), and, if available, provide registration information including registration number. | 6-9  Review protocol explained in detail throughout methods section |
| Eligibility criteria | 6 | Specify study characteristics (e.g., PICOS, length of follow-up) and report characteristics (e.g., years considered, language, publication status) used as criteria for eligibility, giving rationale. | 6  “We excluded newspapers that we either could not fully access using these University of California-subscribed databases or that did not contain articles relevant to our study. We searched the phrases “cancer cell resistance,” “cancer drug resistance,” “tumor drug resistance,” “cancer tumor treatment failure,” “HIV resistance,” “mosquito insecticide resistance,” “plant pesticide resistance,” “weed pesticide resistance,” and “weed herbicide resistance” (excluding “evolve” from all terms to avoid biasing the search), then carefully read each article published between 1980 and 2015 to determine relevancy. We classified articles as relevant if they contained at least one phrase in which it was reasonable to expect use of the word “evolve” in the context of resistance. If the article discussed resistance without explaining that it evolved, it was not considered relevant, as there was no discussion of the development of resistance in which use of the word “evolve” could be expected. We excluded letters to the editor, articles that were less than 100 words, articles that contained multiple disconnected topics unrelated to our search term, and duplicate articles. In the case of duplicate articles, the longer article (if one version had been published with more material) or the more recent article (if they were the same length) was chosen.”  7  “We used the same relevancy criteria [for journals] that were used for the newspapers.”  9  “We looked for cancer in the indexes of the top 10 textbooks that were published after 1976 (when the paper first introducing tumor evolution (Nowell 1976) came out) and only analyzed textbooks with titles that suggested a scope of general/introductory evolution or molecular evolution (S3 Table).” |
| Information sources | 7 | Describe all information sources (e.g., databases with dates of coverage, contact with study authors to identify additional studies) in the search and date last searched. | 6  “Following the methods in Singh et al. (Singh et al. 2016), we identified the top 25 U.S. digital daily newspapers using the Alliance for Audited Media (Lulofs 2013) and accessed them via Newsbank, LexisNexis, Proquest, and EBSCOhost (Table 1).”  7  We used SCImago Journal and Country Rank (SCImago 2007), which determines rank by the number of citations received by a journal and the prestige of the journal where the citation was published, to find the top 5 journals specific to each of the following categories accessed using the Melvyl University of California Catalog: oncology, virology, entomology, and agronomy and crop science (Table 2).”  Page 7 “We further identified different categories of journals that published papers on cancer to examine “evolve” usage by different groups of tumor resistance researchers. We selected widely known journals with high impact factors: 5 general science journals, 5 general clinical journals, 5 cancer-specific journals, and a journal dedicated to drug resistance (Table 2). We applied the same methods as for other journals to select relevant papers.“  8-9  We filtered textbooks by “United States” and “biology” and then the search term “evolution” using The Open Syllabus Project (The American Assembly 2016), which determines which textbooks are most popularly used in publically-accessible college syllabi. We looked for cancer in the indexes of the top 10 textbooks that were published after 1976 (when the paper first introducing tumor evolution (Nowell 1976) came out) and only analyzed textbooks with titles that suggested a scope of general/introductory evolution or molecular evolution (S3 Table).” |
| Search | 8 | Present full electronic search strategy for at least one database, including any limits used, such that it could be repeated. | 6  “We searched the phrases “cancer cell resistance,” “cancer drug resistance,” “tumor drug resistance,” “cancer tumor treatment failure,” “HIV resistance,” “mosquito insecticide resistance,” “plant pesticide resistance,” “weed pesticide resistance,” and “weed herbicide resistance” (excluding “evolve” from all terms to avoid biasing the search), then carefully read each article published between 1980 and 2015 to determine relevancy. We classified articles as relevant if they contained at least one phrase in which it was reasonable to expect use of the word “evolve” in the context of resistance. If the article discussed resistance without explaining that it evolved, it was not considered relevant, as there was no discussion of the development of resistance in which use of the word “evolve” could be expected. We excluded letters to the editor, articles that were less than 100 words, articles that contained multiple disconnected topics unrelated to our search term, and duplicate articles. In the case of duplicate articles, the longer article (if one version had been published with more material) or the more recent article (if they were the same length) was chosen.” |
| Study selection | 9 | State the process for selecting studies (i.e., screening, eligibility, included in systematic review, and, if applicable, included in the meta-analysis). | 6  “We searched the phrases “cancer cell resistance,” “cancer drug resistance,” “tumor drug resistance,” “cancer tumor treatment failure,” “HIV resistance,” “mosquito insecticide resistance,” “plant pesticide resistance,” “weed pesticide resistance,” and “weed herbicide resistance” (excluding “evolve” from all terms to avoid biasing the search), then carefully read each article published between 1980 and 2015 to determine relevancy. We classified articles as relevant if they contained at least one phrase in which it was reasonable to expect use of the word “evolve” in the context of resistance. If the article discussed resistance without explaining that it evolved, it was not considered relevant, as there was no discussion of the development of resistance in which use of the word “evolve” could be expected. We excluded letters to the editor, articles that were less than 100 words, articles that contained multiple disconnected topics unrelated to our search term, and duplicate articles. In the case of duplicate articles, the longer article (if one version had been published with more material) or the more recent article (if they were the same length) was chosen.”  7  “We searched the phrases previously mentioned, limiting our search to 1980-2015 to match the newspaper year range, and then randomly selected 100 papers for each journal to check for relevancy, checking all papers in a journal when our keywords yielded fewer than 100 results. We used the same relevancy criteria that were used for the newspapers.“  8-9  “We filtered textbooks by “United States” and “biology” and then the search term “evolution” using The Open Syllabus Project (The American Assembly 2016), which determines which textbooks are most popularly used in publically-accessible college syllabi. We looked for cancer in the indexes of the top 10 textbooks that were published after 1976 (when the paper first introducing tumor evolution (Nowell 1976) came out) and only analyzed textbooks with titles that suggested a scope of general/introductory evolution or molecular evolution (S3 Table). “ |
| Data collection process | 10 | Describe method of data extraction from reports (e.g., piloted forms, independently, in duplicate) and any processes for obtaining and confirming data from investigators. | 7  “Once we collected the relevant newspaper articles (S1 Table) and papers (S2 Table), we searched for the word “evolve” and its lexemes by using “evol” in the browser search feature and carefully reading the surrounding text to confirm that the word was used in the context of the evolution of resistance. Using the same method, we searched for the substitute words “acquire,” “adapt,” “develop,” “emerge,” and “mutate” and their lexemes. We chose these substitute words based on those found in previous studies (Antonovics et al. 2007; Singh et al. 2016) and a preliminary survey of the data.”  8  “We looked for cancer in the indexes of the top 10 textbooks that were published after 1976 (when the paper first introducing tumor evolution (Nowell 1976) came out) and only analyzed textbooks with titles that suggested a scope of general/introductory evolution or molecular evolution (S3 Table).“ |
| Data items | 11 | List and define all variables for which data were sought (e.g., PICOS, funding sources) and any assumptions and simplifications made. | 7  “Once we collected the relevant newspaper articles (S1 Table) and papers (S2 Table), we searched for the word “evolve” and its lexemes by using “evol” in the browser search feature and carefully reading the surrounding text to confirm that the word was used in the context of the evolution of resistance. Using the same method, we searched for the substitute words “acquire,” “adapt,” “develop,” “emerge,” and “mutate” and their lexemes.”  8  “We compared each newspaper’s HIV drug resistance “evolve” frequency with the HIV incidence rate indicated in the state that its headquarters resided in as reported in the Centers of Disease Control and Prevention’s 2014 HIV Surveillance Report (CDC 2015).”  9  “We looked for cancer in the indexes of the top 10 textbooks that were published after 1976 (when the paper first introducing tumor evolution (Nowell 1976) came out) and only analyzed textbooks with titles that suggested a scope of general/introductory evolution or molecular evolution (S3 Table).“ |
| Risk of bias in individual studies | 12 | Describe methods used for assessing risk of bias of individual studies (including specification of whether this was done at the study or outcome level), and how this information is to be used in any data synthesis. | 7  “Multiple readers assessed each article to confirm relevancy and that “evolve” and the substitute words were used in the appropriate context.” |
| Summary measures | 13 | State the principal summary measures (e.g., risk ratio, difference in means). | N/A |
| Synthesis of results | 14 | Describe the methods of handling data and combining results of studies, if done, including measures of consistency (e.g., I^2^) for each meta-analysis. | 8  “We combined the oncology journals and the additional cancer-specific journals we selected in a single category: cancer-specific (Table 2). We used this category to represent cancer journals for comparisons between categories, and to compare against other categories of cancer journals: general science, general clinical, and the drug resistance specific journal.“ |

Page 1 of 2

| **Section/topic** | **#** | **Checklist item** | **Reported on page #** |
| --- | --- | --- | --- |
| Risk of bias across studies | 15 | Specify any assessment of risk of bias that may affect the cumulative evidence (e.g., publication bias, selective reporting within studies). | 7  “We further identified different categories of journals that published papers on cancer to examine “evolve” usage by different groups of tumor resistance researchers. We selected widely known journals with high impact factors: 5 general science journals, 5 general clinical journals, 5 cancer-specific journals, and a journal dedicated to drug resistance (Table 2).” |
| Additional analyses | 16 | Describe methods of additional analyses (e.g., sensitivity or subgroup analyses, meta-regression), if done, indicating which were pre-specified. | N/A |
| **RESULTS** | | |  |
| Study selection | 17 | Give numbers of studies screened, assessed for eligibility, and included in the review, with reasons for exclusions at each stage, ideally with a flow diagram. | 9  “The number of articles identified during each stage of the screening process is shown in Figure 1.” |
| Study characteristics | 18 | For each study, present characteristics for which data were extracted (e.g., study size, PICOS, follow-up period) and provide the citations. | 9  “For the articles examined, we found that overall “evolve” usages in articles dealing with resistance were 7.9% in newspaper articles and 22.2% in scientific journal papers (Fig. 2), which represented a significant difference (p-value < 0.00001). With regards to specific categories (tumor, HIV, mosquito, weed) for newspapers and journals, “evolve” usage ranged from 3.9% to 52.4% (Fig. 3).”  10  “Finally, we found that 2 out of the 10 evolution textbooks we examined mentioned “cancer” in the index and discussed it in a relevant evolutionary context.” |
| Risk of bias within studies | 19 | Present data on risk of bias of each study and, if available, any outcome level assessment (see item 12). | 10  “Neither newspapers nor journals showed any tendency for “evolve” usage to increase over time for tumor resistance (Pearson’s r: 0.14; p-value: 0.43 and Pearson’s r: 0.03; p-value: 0.87, respectively) (Fig. 7), perhaps because overall usage is so low.” |
| Results of individual studies | 20 | For all outcomes considered (benefits or harms), present, for each study: (a) simple summary data for each intervention group (b) effect estimates and confidence intervals, ideally with a forest plot. | 9  “For the articles examined, we found that overall “evolve” usages in articles dealing with resistance were 7.9% in newspaper articles and 22.2% in scientific journal papers (Fig. 2), which represented a significant difference (p-value < 0.00001). With regards to specific categories (tumor, HIV, mosquito, weed) for newspapers and journals, “evolve” usage ranged from 3.9% to 52.4% (Fig. 3).”  10  “Concerning the cancer tumor drug resistance category, both newspapers and journals used the word “evolve” less frequently in the context of cancer than all of the other categories combined (p-value: 0.01 for newspapers and p-value < 0.00001 for journals) (Fig. 3). Moreover, across the different types of cancer journals, “evolve” usage also differed significantly (p-value: 0.04), with General Science and Drug Resistance category journals using “evolve” with the highest rates at 19.8% and 18.6%, respectively (Fig. 6).”  “Finally, we found that 2 out of the 10 evolution textbooks we examined mentioned “cancer” in the index and discussed it in a relevant evolutionary context.” |
| Synthesis of results | 21 | Present results of each meta-analysis done, including confidence intervals and measures of consistency. | 9-10  “There was, however, a significant difference between the frequencies of “evolve” usage across each category for both newspapers (p-value < 0.00001) and journals (p-value < 0.00001). For newspapers, weed pesticide resistance had the highest “evolve” usage frequency with 25.9%, followed in order by mosquito, HIV, and tumor resistance. For journals, the weed pesticide resistance category again used “evolve” at the highest levels at 52.4%, followed in order by HIV, mosquito, and tumor resistance (Fig. 3). We found that in both newspapers and journals, the substitute words “develop” and “mutate” were used much more frequently than the word “evolve” (Fig. 4). Additionally, while newspapers mostly used only these two words more than “evolve,” journals also used the substitute words “emerge” and “acquire” more often than “evolve.”  In associating the number of relevant articles and “evolve” usage frequency found within the HIV drug resistance category with HIV incidence rates per state, we did not find a significant correlation between number of relevant articles and HIV incidence rates (Pearson’s r: 0.44; p-value: 0.16) or between “evolve” usage and HIV incidence rates (Pearson’s r: -0.59; p-value: 0.04) (Fig. 5).  Concerning the cancer tumor drug resistance category, both newspapers and journals used the word “evolve” less frequently in the context of cancer than all of the other categories combined (p-value: 0.01 for newspapers and p-value < 0.00001 for journals) (Fig. 3). Moreover, across the different types of cancer journals, “evolve” usage also differed significantly (p-value: 0.04), with General Science and Drug Resistance category journals using “evolve” with the highest rates at 19.8% and 18.6%, respectively (Fig. 6).  Neither newspapers nor journals showed any tendency for “evolve” usage to increase over time for tumor resistance (Pearson’s r: 0.14; p-value: 0.43 and Pearson’s r: 0.03; p-value: 0.87, respectively) (Fig. 7), perhaps because overall usage is so low.” |
| Risk of bias across studies | 22 | Present results of any assessment of risk of bias across studies (see Item 15). | N/A |
| Additional analysis | 23 | Give results of additional analyses, if done (e.g., sensitivity or subgroup analyses, meta-regression [see Item 16]). | N/A |
| **DISCUSSION** | | |  |
| Summary of evidence | 24 | Summarize the main findings including the strength of evidence for each main outcome; consider their relevance to key groups (e.g., healthcare providers, users, and policy makers). | 10  “Significant differences in the use of the word “evolve” within different types of scientific journals suggest that the use of evolutionary principles in discussing key issues may vary significantly between fields. Newspapers and journals both used the word “evolve” with different frequencies depending on the topic discussed (Fig. 3). Interestingly, both newspapers and journals used “evolve” most frequently when discussing pesticide resistance in weeds, and least frequently when discussing cancer tumor drug resistance. This may reflect the fact that reporters speak to and read various sources outside of scientific research when writing articles, including doctors, politicians, and industry professionals. These sources also influence the way that reporters choose to frame discourse on the issue (Berkowitz 2009).”  11  “However, our analysis revealed no correlation between number of relevant articles and HIV incidence, or between “evolve” usage and HIV incidence. In fact, “evolve” usage was low across all states (Fig. 5).”  12  “But regardless of this gap between the general public and scientific audiences, we found that the word “evolve” was used significantly less frequently when discussing cancer tumor drug resistance than all other topics in both newspapers and journals (3.9% and 9.8%, respectively) (Fig. 3).”  “Furthermore, we found no trend of increasing usage of "evolve" over time in newspaper or journal cancer categories (Pearson correlations, p-values: 0.43 and 0.87, respectively).” |
| Limitations | 25 | Discuss limitations at study and outcome level (e.g., risk of bias), and at review-level (e.g., incomplete retrieval of identified research, reporting bias). | 11  “It is important to note that simply using the word “evolve” does not indicate that the audience reading the article will understand evolution. Rather, we suggest that overall usage of the word “evolve” (for example, in a field or journal) can be a rough indicator of overall recognition of evolutionary processes. Use of “evolve” in newspapers and journals informs us about the way discussions about certain topics are framed rather than reflecting understanding or acceptance of those topics. Indeed, there is likely a large gap between how academics understand evolution and how the general public understands evolution (Nadelson & Sinatra 2010; Nisbet & Mooney 2009; Spiegel et al. 2006). The science-society relationship is complex and does not rely solely on a top-down flow of information. It is complicated by multiple factors determining the way that the public reacts to this information (Nisbet & Mooney 2009).” |
| Conclusions | 26 | Provide a general interpretation of the results in the context of other evidence, and implications for future research. | 12-14  “Our finding that “evolve” was used in the drug-resistance journal and general science journals significantly more often than in the general clinical and cancer-specific journals (Fig. 6) could further reflect how evolutionary concepts are infrequently introduced and slow to spread in the medical field (Antonovics et al. 2007; Nesse & Stearns 2008). While ecology and evolution journals cite medical journals occasionally, medical journals cite ecology and evolution journals rarely. This suggests an asymmetry in the relationship between medicine and evolutionary biology (Nesse & Stearns 2008; Rosvall & Bergstrom 2008). If healthcare professionals are less exposed to evolutionary principles, it could prevent them from using evolutionary perspectives in understanding and dealing with problems such as cancer tumor drug resistance. This is an area for investigation.  Indeed, the lower rate of “evolve” usage we found in journals with a more medical audience (general clinical and cancer-specific) may be reflective of a lack of recognition of evolution as an important concept for medical consideration. Most medical schools do not have evolutionary biologists on the faculty (Nesse & Stearns 2008), and many medical students do not accept the theory of evolution (Nesse & Stearns 2008). This may be due to the common conception that a clinician’s knowledge of the origins and evolution of pathogens is often not essential in administering successful treatments to patients (MacCallum 2007), or it could be due to other reasons that we currently do not understand. However, increasing exposure to evolutionary concepts can be valuable in the overarching treatment of medical problems (Nesse et al. 2010; Nesse & Stearns 2008; Stearns 2012; Stearns et al. 2010). Not only can research on the evolutionary reasons behind clinical occurrences potentially help shape treatment options, but evolution can provide a framework for organizing medical knowledge more generally (Nesse & Stearns 2008).  Ultimately, although researchers demonstrate a greater tendency to use evolutionary language compared with journalists, the use of the word “evolve” was consistently low for both groups, especially in comparison to substitute words (Fig. 4). Different frequencies of “evolve” usage depending on category (HIV drug resistance, tumor resistance, etc.) and type of journal discussing cancer tumor drug resistance could reflect discrepancies in recognition of resistance problems as evolutionary issues across fields (Antonovics et al. 2007). These discrepancies could be preventing certain fields from applying evolutionary theory to combat resistance issues or from recognizing the applicability of solutions to analogous issues in other fields (Palumbi 2001).  There may be many reasons for why an author does not use the word “evolve.” However, using the word “evolve” instead of unclear or less accurate alternatives when discussing important evolutionary issues may help make the topic clearer to readers. In addition to increasing exposure to the theory of evolution, which may be downplayed due to low public acceptance (Scott & Branch 2003), using the word “evolve” when discussing topics like tumor resistance and weed pesticide resistance may help shape the way that the general public, clinicians, and scientists in different fields think about and approach these topics.” |
| **FUNDING** | | |  |
| Funding | 27 | Describe sources of funding for the systematic review and other support (e.g., supply of data); role of funders for the systematic review. | Entered in PeerJ submission system (N/A) |

*From:*  Moher D, Liberati A, Tetzlaff J, Altman DG, The PRISMA Group (2009). Preferred Reporting Items for Systematic Reviews and Meta-Analyses: The PRISMA Statement. PLoS Med 6(7): e1000097. doi:10.1371/journal.pmed1000097

For more information, visit: **www.prisma-statement.org**.

Page 2 of 2
